# Lightweight 2.5D SLAM with Dynamic Map Refinement and Height-Aware Encoding for Resource-Constrained Indoor Robots

**DOI:** 10.3390/s26154765

**Published:** 2026-07-27

**Authors:** Guitao Yu, Yuping Zhang, Zhiao Qi, Kui Yang, Yang He, Dongtai Liang

**Affiliations:** 1 School of Automation Engineering, University of Electronic Science and Technology of China, Chengdu 611731, China; 2 Healthy & Intelligent Kitchen Engineering Research Center of Zhejiang Province, Bin-Hai 6th Road, Ningbo 315336, China or heyangc@fotile.com (Y.H.); liangdongtai@nbu.edu.cn (D.L.); 3 Faculty of mechanical Engineering and Mechanics, Ningbo University, Ningbo 315211, Chinayangkui0108@163.com (K.Y.)

**Keywords:** SLAM, multi-sensor fusion, embedded system, 2.5D mapping, dynamic artifact removal, height-aware occupancy map

## Abstract

Indoor mobile robots equipped with low-cost and sparse sensors often suffer from limited vertical perception and dynamic residual artifacts in the final map. This paper presents a lightweight 2.5D simultaneous localization and mapping (SLAM) framework using a single-line laser distance sensor (LDS), time-of-flight (ToF) sensing, wheel odometry, and an inertial measurement unit (IMU). In this work, 2.5D refers to a 2D grid map with discretized vertical occupancy bins for each grid cell, rather than a full continuous 3D reconstruction. The system integrates multi-sensor synchronization, motion correction, error-state Kalman filter (ESKF)-based state estimation, normal distributions transform (NDT) registration, and pose graph optimization to reconstruct a pose-consistent global map. Based on this map, an offline dynamic refinement module estimates temporal voxel support across keyframes, extracts low-support candidate regions, and applies geometric clustering and isolated-point filtering to suppress transient residual artifacts while preserving stable structures. A 24-bit RGB occupancy encoding is further proposed to store the discretized vertical occupancy state in a compact three-channel image format. The proposed framework emphasizes system-level deployment value by combining sparse multi-sensor mapping, conservative offline refinement, and compact height-aware map export on a low-cost indoor robot platform. Experiments on public datasets, embedded hardware, and self-collected indoor sequences evaluate odometry reference performance, resource usage, platform-specific 2.5D mapping, dynamic refinement, and height-aware encoding.

## 1. Introduction

The field of autonomous mobile robotics has experienced significant advancements over recent decades, driven by advances in hardware and algorithmic development. Effective navigation relies on three essential components: localization, path planning, and control. In unknown environments, autonomous robots lack prior knowledge of their surroundings. Simultaneous localization and mapping (SLAM) solves this problem by enabling robots to construct maps and estimate their poses without pre-existing environmental information [1,2]. SLAM systems leverage a diverse array of sensor modalities. LiDAR-based SLAM utilizes laser scanners to acquire high-precision range measurements, while visual SLAM employs monocular, stereo, or RGB-D cameras to extract visual features for state estimation.

In recent years, high-performance SLAM algorithms have made significant progress. HectorSLAM utilizes laser scanners without relying on odometry and achieves robust performance through efficient scan matching [3]. Similarly, the classic Gmapping algorithm combines LiDAR and odometry using a Rao-Blackwellized particle filter to achieve accurate 2D mapping [4]. Although these algorithms perform well in 2D map construction, they are limited in complex indoor environments because conventional 2D maps discard vertical information. Full 3D LiDAR SLAM can capture richer spatial structures and provide more complete environmental perception, as demonstrated by representative LiDAR and LiDAR-inertial SLAM systems such as LOAM, LeGO-LOAM, LIO-SAM, and FAST-LIO2 [5,6,7,8]. However, 3D LiDAR sensors are expensive and impose high computational and storage requirements, making them difficult to deploy on resource-constrained embedded platforms.

With the rapid expansion of the consumer robotics market, the demand for SLAM technology in consumer-grade robots is steadily increasing, especially on embedded platforms. Due to limited memory, computing power, and energy consumption, designing and implementing SLAM systems on such platforms remains challenging. Algorithm execution often requires substantial processing resources, which may cause response latency and reduce real-time performance on low-power embedded devices. Therefore, a practical indoor SLAM system should balance sensing cost, mapping accuracy, computational efficiency, and map representation capability [9,10].

To address these challenges, this paper proposes a lightweight 2.5D SLAM framework for indoor robots equipped with low-cost and sparse sensors. The system integrates single-line LiDAR, ToF sensing, wheel odometry, and IMU measurements, following the general trend of multi-sensor fusion for robust indoor mobile robot perception [9,10,11]. Compared with traditional 2D mapping, the proposed framework preserves partial vertical information. Compared with full 3D SLAM, it reduces data processing and storage requirements, making it suitable for resource-constrained embedded platforms. The multi-sensor fusion pipeline includes timestamp alignment, motion distortion correction, ESKF-based state correction, NDT-based scan-to-map registration, and pose graph optimization, providing the foundation of the system.

However, in practical indoor mapping, the final map is affected not only by sparse vertical perception but also by dynamic residual artifacts. Pedestrians, footprints, temporary obstacles, and short-term ToF reflections may remain in the accumulated global map. Similar dynamic-object and transient-residual problems have been widely discussed in recent dynamic SLAM and LiDAR filtering studies [12,13,14,15]. These residual points usually have limited influence on pose optimization, but they degrade the readability and usability of the final occupancy map. Therefore, this work further introduces an offline dynamic map refinement module to improve the quality of the final map. The module estimates temporal voxel support across keyframes, extracts low-support candidate regions, and combines geometric clustering with isolated-point filtering to suppress transient residual artifacts while preserving stable structures.

Several map representations have been used for mobile robot mapping. Standard 2D occupancy grids represent the environment as planar occupied or free cells and are widely used for indoor navigation because of their simplicity, compactness, and compatibility with planning systems [16]. However, they discard vertical occupancy information. Voxel and octree-based maps, such as OctoMap, preserve richer 3D occupancy, but they usually require higher memory usage, update cost, and serialization complexity [17]. Elevation maps and 2.5D maps extend planar grids by associating height information with each grid cell, enabling partial vertical structure representation without constructing a full 3D model [18,19,20]. Multi-layer grids can further represent several vertical layers per planar cell and are useful for discontinuous or overhanging structures. NDT-based maps model local point distributions probabilistically and are useful for registration and map matching [21,22], but they are not primarily designed as compact image-compatible height-bin export formats. Motivated by this trade-off, this work uses a 2D grid with a 24-bin vertical occupancy vector for each cell and packs the discretized height-bin occupancy into a three-channel 24-bit image. This representation does not aim to replace full 3D occupancy maps; instead, it provides a compact and directly decodable export format for sparse height-aware indoor mapping.

Classical components such as ESKF, NDT registration, keyframe selection, motion correction, and pose graph optimization are used as the SLAM backbone in this work. The novelty of this work is therefore positioned at the system level: adapting mature SLAM modules to a low-cost sparse-sensing indoor robot, conservatively refining residual artifacts after global map fusion, and exporting a compact height-aware map representation for deployment. Therefore, this work does not claim a new general-purpose SLAM optimizer or a new dynamic SLAM theory. Instead, it focuses on a deployable system configuration for sparse low-cost indoor robots.

The main contributions of this paper are summarized as follows:

1. A lightweight multi-sensor 2.5D mapping pipeline is developed for resource-constrained indoor robots equipped with a single-line LDS, sparse ToF sensing, wheel odometry, and IMU measurements. Mature SLAM modules, including synchronization, motion correction, ESKF, NDT registration, keyframe selection, and pose graph optimization, are adapted to generate a pose-consistent sparse global map on a low-cost platform.

2. A conservative offline dynamic map refinement strategy is introduced for the accumulated global map. The module uses temporal voxel support, sensor-source support, geometric clustering, and isolated-point filtering to suppress low-support residual artifacts while protecting stable LDS-supported structures.

3. A compact 24-bit height-aware occupancy encoding is developed as an image-compatible export format. Each 2D grid cell stores a 24-bin vertical occupancy vector packed into three 8-bit channels, enabling bitwise recovery of discretized height occupancy rather than continuous 3D point reconstruction.

4. Experiments are conducted using public datasets, embedded hardware tests, and self-collected indoor robot sequences. Public datasets are used for odometry reference evaluation, while self-collected sequences evaluate platform-specific mapping, dynamic refinement, parameter sensitivity, and 24-bit height-aware map export.

## 2. Materials and Methods

### 2.1. Hardware Platform and Experimental Data

**Figure 1:**
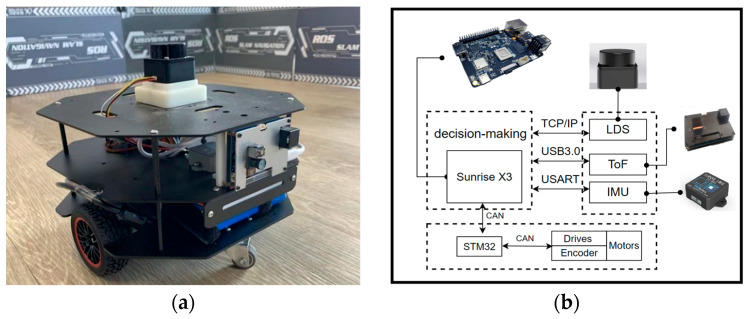
Hardware equipment. (**a**) The robot platform for the experiment; (**b**) hardware connection method.

The experimental platform uses an indoor mobile robot equipped with a low-cost and sparse sensor configuration. The decision layer is powered by a Sunrise X3 embedded processor with a quad-core ARM Cortex-A53 CPU, 4 GB memory, and 5 TOPS computing capability. The sensing system consists of an STL-06p LDS sensor for 360-degree planar range measurements, a Cleaner01F1 linear ToF module for sparse vertical observations, an A9 IMU, and wheel odometry for motion estimation and state correction (Figure 1).

The self-collected indoor sequences are divided into two groups. The first group consists of standard indoor mapping scenes, including the IEC/ASTM 62885-7 coverage test-bed scene and the household environment scene, which are used for basic map consistency evaluation. The second group consists of dynamic indoor loop sequences, including a long-loop sequence and a short-loop sequence collected in the same loop scene. The nearly static IEC/ASTM 62885-7 coverage test-bed scene is additionally used for conservative cross-scene validation.

### 2.2. System Overview

**Figure 2:**
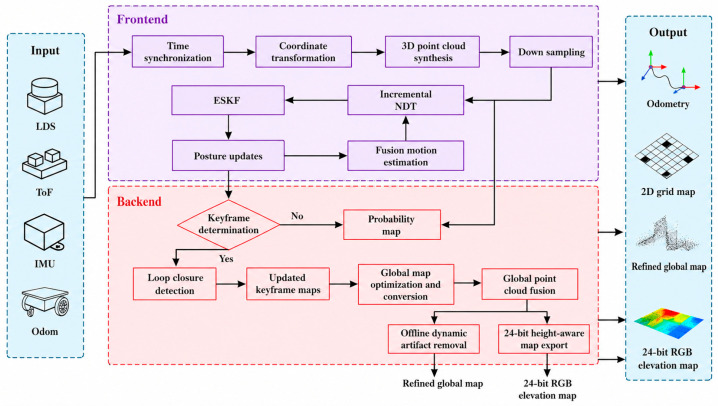
Overall framework of the proposed multi-sensor SLAM system. After global point cloud fusion, the framework performs offline dynamic artifact removal and 24-bit height-aware map export as two post-processing branches.

The proposed system consists of a front-end multi-sensor fusion module, a back-end optimization module, and a map generation module. In the front-end, data from LDS, ToF, IMU, and wheel odometry are synchronized and fused to ensure temporal and spatial consistency. Incremental NDT provides pose estimates for point cloud registration, while the ESKF corrects state errors in real time using multi-sensor measurements. In the back-end, pose graph optimization and loop closure detection are used to correct accumulated drift and improve global consistency. Finally, the system outputs optimized odometry, a fused global point cloud, a standard 2D occupancy grid, and a 24-bit height-aware 2.5D map. When offline refinement is enabled, a refined global point cloud is further generated for final map export (Figure 2).

In addition to the online keyframe-level mapping pipeline, two post-processing branches are introduced after global map fusion: offline dynamic artifact removal and 24-bit height-aware occupancy map export. These two branches improve the usability and compactness of the final map without changing the online front-end odometry process.

The front-end odometry and keyframe-level backend update are executed during data collection, whereas dynamic artifact removal and 24-bit height-aware map export are executed after global map fusion.

### 2.3. Sensor Synchronization and Preprocessing

The preprocessing module includes timestamp alignment, keyframe selection, and motion distortion correction. These steps convert asynchronous multi-sensor measurements into temporally aligned and spatially corrected keyframe point clouds for NDT registration, global map construction, dynamic artifact removal, and height-aware map encoding.

#### 2.3.1. Timestamp Alignment

**Figure 3:**
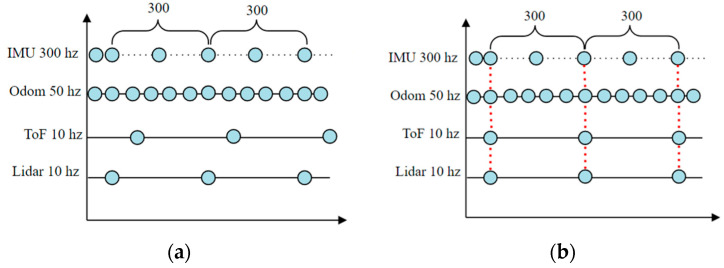
Data alignment. (**a**) before timestamp alignment; (**b**) after timestamp alignment.

Multi-sensor measurements are acquired asynchronously because the LDS, ToF, IMU, and wheel odometry operate at different sampling rates. To fuse these measurements at the frame level, the LDS scan timestamp is used as the reference time, and the measurements from the other sensors are selected or interpolated around this timestamp. Figure 3 illustrates the timestamp alignment process before and after synchronization.

For the *k*-th LDS scan, the timestamp matching condition is expressed as Equation (1).

(1) $$|t_{LDS,k}-t_{s,j}|\leq \tau_{sync},\;s\in \{ToF,IMU,Odom\},$$

where $t_{LDS,k}$ denotes the timestamp of the *k*-th LDS scan, $t_{s,j}$ denotes the timestamp of the nearest measurement from sensor s, and $\tau_{sync}$ is the maximum allowed synchronization threshold.

If two adjacent measurements of sensor s are available around tLDS, k, the corresponding measurement is interpolated as Equation (2).

(2) $$z(t_{LDS,k})=\mathrm{Interp}\left(z(t_{i}),z(t_{i+1}),t_{LDS,k}\right)$$

where $z_{s}(t_{i})$ and $z_{s}(t_{i+1})$ are two adjacent measurements of sensor s, and $z_{s}(t_{LDS,k})$ is the synchronized measurement used by the frontend estimator and point cloud fusion module.

This formulation defines the synchronization window and interpolation process used to align asynchronous multi-sensor measurements.

#### 2.3.2. Keyframe Selection

**Figure 4:**
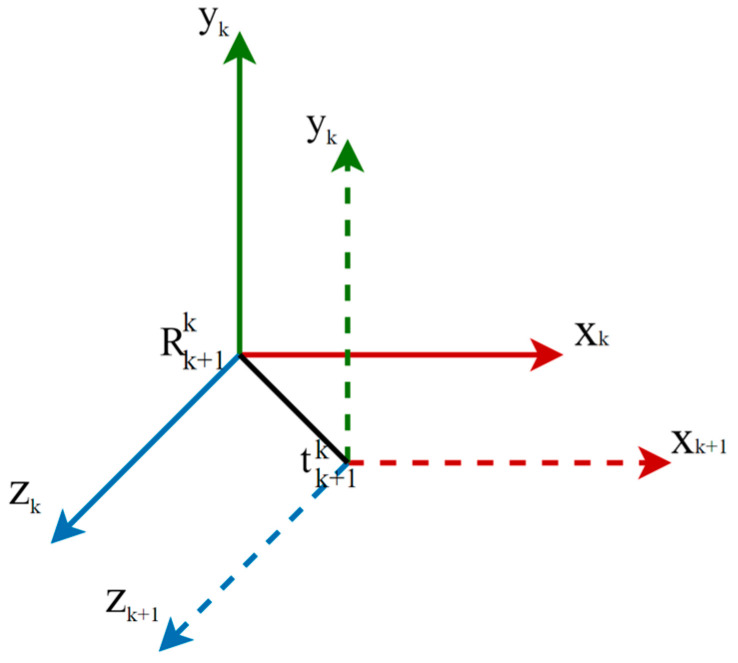
Pose transformation.

To reduce redundant computation on the embedded platform, a motion-based keyframe selection mechanism is used. Keyframes are selected according to the motion change between the current frame and the latest keyframe, as illustrated in Figure 4:

The k and k+1 represent the latest keyframe and the current frame, and $R_{k+1}^{k}$ and $t_{k+1}^{k}$ represent the rotation and translation between the latest keyframe and the current frame. The criteria for determining a keyframe are as follows:

(1) The time interval between k and k+1 is greater than 0.3 s;

(2) The translation distance $t_{k+1}^{k}$ is greater than 0.15 m;

(3) The corresponding rotation angle $R_{k+1}^{k}$ is greater than 10°.

In this work, the selected keyframes are also used as temporal observation units for dynamic artifact removal. Therefore, the keyframe selection strategy directly affects the temporal voxel support statistics used in the offline map refinement module.

#### 2.3.3. Motion Distortion Correction

The actual sampling frequency of LiDAR is so high that it is usually impossible to process each new point as it is received. As a result, point clouds are typically accumulated over a period of time. The set of accumulated points is referred to as a frame, which also serves the purpose of frequency down-sampling. If the LiDAR is in motion during this period, the positions of the 3D points within the frame will be inconsistent due to motion distortion. Therefore, the IMU state estimation results are used to interpolate and correct this distortion, restoring the point cloud to its actual spatial position. The mathematical model is defined as Equation (3).

(3) $${\hat{\dot{P}}}_{b_{t}}=R_{I}^{L}\left(R_{W}^{b_{k+1}}\left(\left(R_{b_{t}}^{W}\left(R_{L}^{I}{\hat{P}}_{b_{t}}+t_{L}^{I}\right)+t_{b_{t}}^{W}\right)-t_{b_{k+1}}^{W}\right)-t_{L}^{I}\right)$$

where $R_{I}^{L}$ and $t_{L}^{I}$ represent the rotation and translation of the LiDAR relative to the IMU coordinate system, respectively. ${\hat{\dot{P}}}_{b_{t}}$ are the 3D point coordinates under the LiDAR coordinate system corresponding to the IMU data frame in $\left[t_{k},t_{k+1}\right]$, and ${\hat{\dot{P}}}_{b_{t}}$ is the 3D coordinate after removing the distortion.

First, ${\hat{\dot{P}}}_{b_{t}}$ is transformed to the IMU coordinate system at the current moment, and then it is converted to the world coordinate system using the pre-integrated estimated pose estimates $R_{b_{t}}^{W}$, $t_{b_{t}}^{W}$. Then it is converted to the IMU coordinate system corresponding to the end moment of the LiDAR frame. Finally, it is converted to the LiDAR coordinate system to obtain the point cloud after removing the distortion.

The corrected keyframe point clouds are later used for NDT registration, global map fusion, temporal voxel support estimation, and 24-bit height-aware occupancy encoding.

#### 2.3.4. Preprocessing Output for Map Refinement

After timestamp alignment, keyframe selection, and motion distortion correction, each valid keyframe is stored as a point cloud associated with its optimized pose. In the proposed system, these keyframe-level records are used for incremental NDT registration and global map construction, and are further reused by the offline dynamic artifact removal module to estimate temporal voxel support and by the 24-bit height-aware map export module to encode vertical occupancy.

### 2.4. The Error State Kalman Filter

In the front-end state estimation module, the error-state Kalman filter (ESKF) is used to fuse IMU measurements with motion constraints from wheel odometry and LiDAR registration [23,24]. In ESKF, there are three types of state variables: the true state $X_{t}$, the nominal state $X_{n}$, and the error state δX. The nominal state is propagated according to the system motion model and accumulates errors caused by sensor noise and model imperfections, while the error state captures these deviations. Their relationship is defined as Equation (4).

(4) $$X_{t}=X_{n}⊕\delta X$$

The ESKF process in this paper consists of two steps: prediction and correction. When IMU measurements are received, the nominal state is first updated. Then, the error state is estimated and used to correct the nominal state through the calibration process. By repeating this process, the ESKF continuously optimizes the system state estimation and provides a stable motion prior for incremental NDT registration and backend map optimization.

### 2.5. Backend Optimization and Incremental NDT Mapping

**Figure 5:**
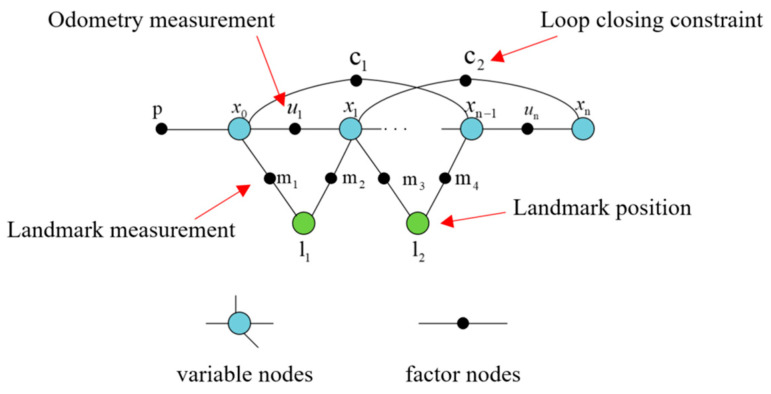
Pose factor map.

Factor graph optimization is widely used in SLAM to represent poses and constraints in a unified graph structure [25]. It can effectively infer the probability distribution of unobserved variables and predict the solution of the problem. In this study, the pose difference is regarded as an optimization variable to better adapt the loop factor. As shown in Figure 5, the self-state $x_{i}$ and observed landmarks $l_{i}$ are used as nodes, motion states $u_{i}$ and observations $m_{i}$ are used as factors. Finally, the loop closure constraint $c_{i}$ is used for correction.

The loop factor in this paper is expressed as Equation (5):

(5) $$f(x_{i},x_{j})=z_{ij}⊖(x_{i}^{-1}⊕x_{j})$$

where $z_{ij}$ denotes the observed relative pose from *i* to *j*, $x_{i}$ and $x_{j}$ denote keyframe poses, and the operator denotes pose composition or pose difference.

For the loop closure factor, nonlinear least squares optimization is used to iteratively adjust the pose variables by minimizing the total error, as shown in Equation (6).

(6) $$E=\underset{(i,j)\in \mathrm{loops}}{\Sigma }{||f(x_{i},x_{j})||}_{\Sigma_{ij}}^{2}$$

where $\Sigma_{ij}$ is the covariance matrix of the measurement noise, indicating the uncertainty of the relative pose constraint. Through this optimization, loop closure constraints help correct accumulated drift and improve the global consistency of the map.

To reduce repeated map rebuilding and improve processing efficiency on the embedded platform, the system uses an incremental NDT-based front-end-to-back-end optimization strategy. The incremental NDT module follows the normal distributions transform representation, in which the point cloud is divided into voxels and the point distribution in each voxel is modeled using a Gaussian distribution [21,22]. When new point cloud data are inserted, the Gaussian parameters of the affected voxels are updated incrementally instead of rebuilding the entire NDT map, as shown in Equations (7) and (8).

(7) $$\mu =\frac{m\cdot \mu_{H}+n\cdot \mu_{A}}{m+n}$$

(8) $$\Sigma \;=\;\frac{1}{m+n}\left[m\cdot \Sigma_{H}+n\cdot \Sigma_{A}+\frac{m\cdot n}{m+n}(\mu_{H}-\mu_{A}){\left(\mu_{H}-\mu_{A}\right)}^{⊤}\right]$$

where m and n denote the numbers of historical and newly added points, respectively; $\mu_{H}$ and $\mu_{A}$ denote their corresponding means; $\Sigma_{H}$ and $\Sigma_{A}$ denote their covariance matrices. These incremental update equations allow the NDT map to be updated efficiently while preserving the statistical structure of the local point cloud distribution.

Assuming that the initial pose estimate of the *k*-th frame is $T_{0}$, and the pose increment relative to the previous frame is $\Delta T_{k}$, the pose of the *k*-th frame is expressed as Equation (9).

(9) $$T_{k}=T_{k-1}\Delta T_{k}$$

The backend further optimizes keyframe poses by minimizing the residual between keyframe point observations and the NDT model, as shown in Equation (10).

(10) $$\arg\;\min\overset{n}{\underset{i=1}{\Sigma }}{\left(z_{i}-h_{NDT}(T_{k},T_{k-1},z_{i})\right)}^{2}\Sigma_{i}$$

where $z_{i}$ is an observation point, $h_{NDT}(T_{k},T_{k-1},z_{i})$ denotes the prediction under the NDT model, and $\Sigma_{i}$ is the covariance matrix associated with the observation point.

The backend optimization and incremental NDT mapping described above generate the optimized keyframe poses and the fused global point cloud map. The optimized global map is further used as the input of two post-processing modules: offline dynamic artifact removal and 24-bit height-aware map export. Therefore, the backend module provides a pose-consistent map foundation for the proposed map refinement and compact height-aware representation.

### 2.6. 2.5D Map Representation and 24-Bit Height-Aware Occupancy Encoding

This design follows the general idea of using compact grid-based map representations to balance spatial expressiveness and computational efficiency [16,17,18,19,20]. In this representation, each 2D grid cell corresponds to a vertical column divided into 24 discrete height bins. Each bit in the three-channel value represents the occupancy state of one height bin, allowing the occupied bin indices to be decoded from the saved image. The corresponding metric height intervals can be recovered using the stored vertical bounds $z_{\min}$ and $z_{\max}$. Therefore, the encoded image is treated as a decodable map representation rather than only a pseudo-color visualization.

#### 2.6.1. 2.5D Height-Bin Grid Construction

The optimized fused global point cloud is projected into a 2D planar grid with horizontal resolution Δxy. Different from a full 3D voxel grid, the proposed 2.5D representation assigns a fixed-length vertical occupancy vector to each 2D grid cell. Each vertical vector contains 24 binary height bins, which can later be packed into a 24-bit image code.

**Figure 6:**
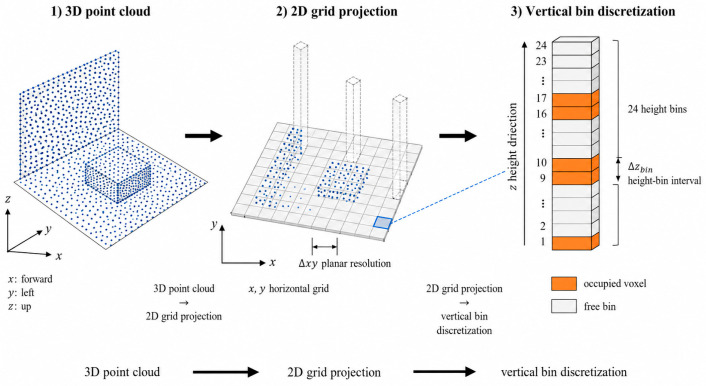
Construction of the 2.5D height-bin grid from the fused global point cloud. Each 2D grid cell corresponds to a vertical column divided into 24 discrete height bins.

The map is organized as a 2D grid, while the vertical occupancy of each grid cell is stored as a 24-bin bitmask (Figure 6).

The 2D grid is defined as Equation (11).

(11) $$G=\{G(i,j)|0\leq i<N_{x},\;0\leq j<N_{y}\},$$

where each grid cell G(i,j) maintains a 24-bin vertical occupancy vector:

(12) $$G(i,j)=\left[b_{0}(i,j),b_{1}(i,j),\ldots ,b_{23}(i,j)\right],\;b_{k}(i,j)\in \{0,1\}.$$

Here in Equation (12), $b_{k}(i,j)=1$ indicates that the k-th height bin of grid cell (i,j) is occupied by at least one point; otherwise, $b_{k}(i,j)=0$.

The planar grid dimensions are computed as Equation (13).

(13) $$N_{x}=\left\lceil \frac{x_{\max}-x_{\min}}{\Delta xy}\right\rceil ,\;N_{y}=\left\lceil \frac{y_{\max}-y_{\min}}{\Delta xy}\right\rceil .$$

where $x_{\min}$, $x_{\max}$, $y_{\min}$, and $y_{\max}$ denote the horizontal boundary of the global point cloud, and Δxy denotes the planar grid resolution.

For a point (x,y,z), its planar grid index is computed as Equation (14).

(14) $$i=\left\lfloor \frac{x-x_{\min}}{\Delta xy}\right\rfloor ,\;j=\left\lfloor \frac{y-y_{\min}}{\Delta xy}\right\rfloor .$$

The vertical height range is uniformly divided into 24 bins. Therefore, the height-bin index is computed as Equation (15).

(15) $$k=\mathrm{clip}\left(\left\lfloor 24\cdot \frac{z-z_{\min}}{z_{\max}-z_{\min}}\right\rfloor ,0,23\right).$$

where $z_{\min}$ and $z_{\max}$ denote the vertical boundary of the global point cloud. The clipping operation ensures that the computed bin index remains within the valid range [0,23], especially for points located at the upper boundary.

The 24 binary height occupancy values of each grid cell are packed into a 24-bit bitmask and stored using three 8-bit image channels. Unlike downward-filled elevation maps, the proposed representation sets a height-bin bit to one only when the corresponding height interval is directly occupied by observed points. Therefore, suspended or discontinuous vertical structures can be preserved instead of being converted into solid columns. The encoded three-channel image is treated as a data image rather than a color visualization.

#### 2.6.2. 24-Bit Height-Aware Occupancy Encoding

The 24 height bins of each vertical column associated with a 2D grid cell are encoded into three 8-bit image channels. Since OpenCV stores color images in BGR order, bins 0–7, 8–15, and 16–23 are assigned to the B, G, and R channels, respectively. Each bit records the occupancy state of one height bin. Therefore, the encoding is reversible for the discretized 24-bin binary occupancy vector, but it does not preserve the original continuous 3D point coordinates.

**Figure 7:**
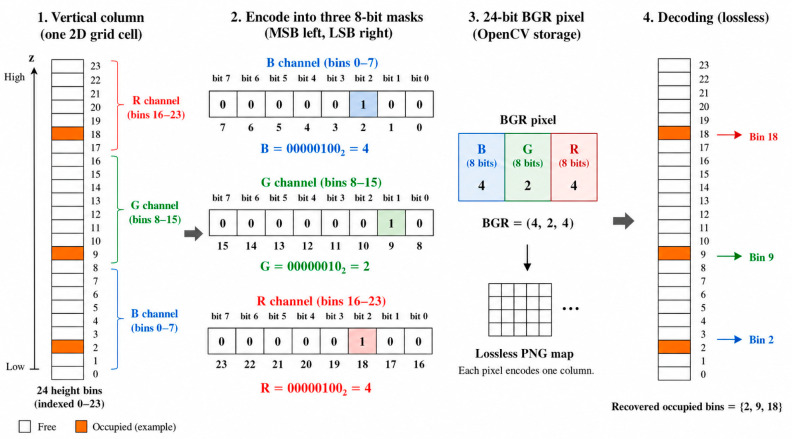
The 24-bit height-aware occupancy encoding. Each 2D grid cell is discretized into 24 vertical height bins indexed from 0 to 23. The bins are encoded into three 8-bit channels: bins 0–7 in B, bins 8–15 in G, and bins 16–23 in R. For the occupied bins {2, 9, 18}, the corresponding bits are B bit 2, G bit 1, and R bit 2, resulting in the OpenCV BGR value (4, 2, 4). The encoded pixel stores the discretized 24-bin occupancy pattern and can be decoded back to the corresponding occupied height bins.

The proposed 24-bit map is interpreted as a decodable occupancy representation rather than a pseudo-color visualization image. Each bit corresponds to a specific height bin, and the occupied height layers of each 2D grid cell can be recovered by bitwise decoding (Figure 7).

The B, G, and R channel values of grid cell (i,j) are computed as Equation (16).

(16) $$\left\{\begin{array}{l} B(i,j)=\sum_{k=0}^{7}b_{k}(i,j)2^{k}\\ G(i,j)=\sum_{k=8}^{15}b_{k}(i,j)2^{k-8}\\ R(i,j)=\sum_{k=16}^{23}b_{k}(i,j)2^{k-16} \end{array}\right.$$

where B(i,j), G(i,j), and R(i,j) denote the 8-bit encoding values of the three image channels, and $b_{k}(i,j)$ denotes the occupancy state of the *k*-th height bin in grid cell (i,j).

The final 24-bit value of grid cell (i,j) is obtained as Equation (17).

(17) $$C(i,j)=B(i,j)\;|\;\left(G(i,j)\ll 8\right)\;|\;\left(R(i,j)\ll 16\right),$$

where ≪ denotes the bitwise left shift operation, and | denotes the bitwise OR operation.

The occupied height bins can be decoded from C(i,j) by checking the corresponding bits. For example, if the bit corresponding to bin k is one, the *k*-th height interval of grid cell (i,j) is considered occupied. This encoding allows the discretized vertical occupancy states to be stored compactly in a three-channel PNG image. Thus, the saved PNG can be decoded back to the same 24-bin occupancy state after discretization.

In the implementation, the encoded 24-bit map is saved as an 8-bit three-channel PNG image without lossy compression, normalization, palette conversion, gamma correction, or color-space conversion. The map boundary, origin, planar resolution, vertical bounds, bin number, coordinate frame, BGR channel order, and bit-to-height-bin mapping are stored consistently with the 2D occupancy grid map, ensuring spatial alignment between the standard occupancy map and the height-aware map.

### 2.7. Offline Dynamic Artifact Removal

After global point cloud fusion, transient objects such as pedestrians, footprints, and short-term ToF reflections may remain as residual artifacts in the final map, similar to the dynamic-map contamination problem discussed in dynamic SLAM and LiDAR filtering studies [12,13,14,15]. To improve map readability, a lightweight offline dynamic artifact removal module is added after global map generation.

This module is designed as a conservative offline post-processing refinement for residual artifacts in the fused global map, rather than as a general online dynamic-object SLAM method. A point cluster is removed only when both low temporal support and geometric artifact constraints are satisfied, while stable LDS-supported structures are protected to reduce false removal.

The module estimates temporal voxel support from the stored keyframe point clouds. Each keyframe cloud is transformed into the world frame using its optimized pose, and each voxel is counted at most once per keyframe if it is observed in that keyframe.

The temporal support of voxel q is defined as Equation (18).

(18) $$S(q)=\sum_{k=1}^{N}I\left(q\in V_{k}\right),$$

where N is the number of keyframes, $V_{k}$ is the set of voxels observed in the *k*-th keyframe, and I(⋅) is the indicator function.

Voxels with low temporal support are treated as candidate dynamic regions, while voxels with stable LDS support are retained to reduce the risk of removing static structures. The refinement strategy is designed conservatively: stable LDS-supported voxels are protected, and a cluster is removed only when temporal inconsistency and geometric artifact constraints are satisfied simultaneously.

Low-support candidate points are clustered using Euclidean clustering. The geometric artifact constraints include cluster size, horizontal span, vertical span, low-support ratio, ToF-only ratio, and stable LDS-support ratio. In the full mode, sparse isolated low-support points near the robot trajectory are further removed using stricter thresholds.

The refined global map is then used for final point cloud data (PCD) saving, 2D occupancy grid generation, and 24-bit height-aware map export.

For ablation analysis, four cleanup modes are implemented: no cleanup, geometry-only filtering, temporal support with geometric filtering, and the full method (Table 1).

## 3. Results

### 3.1. Odometry Reference Experiments on Public Datasets

In this study, the public datasets M2DGR and KITTI [26,27] are used as odometry reference experiments rather than direct benchmarks for the proposed low-cost sparse-sensing platform. Their sensor configurations, motion conditions, and application scenarios differ from the proposed LDS–ToF–wheel odometry–IMU system; therefore, these experiments mainly evaluate whether the adopted SLAM backbone can provide usable trajectory estimation under general ground-robot motion conditions. Platform-specific 2.5D mapping, dynamic refinement, and 24-bit height-aware encoding are evaluated using the self-collected indoor robot sequences. Classical 2D SLAM systems such as Gmapping, HectorSLAM, and Cartographer 2D are important planar-mapping baselines, but they do not preserve vertical occupancy information or perform residual-artifact refinement. Therefore, they are considered planar occupancy-grid references rather than direct baselines for the proposed height-aware representation.

For the public-dataset reference experiments, the absolute trajectory error (ATE) is computed from the Euclidean distance between the estimated and reference positions after timestamp association using the same evaluation script for all compared methods. RMSE denotes the square root of the mean squared ATE, and MEAN denotes the arithmetic mean of the ATE samples; therefore, both metrics are computed from the same set of non-negative trajectory-error samples.

**Figure 8:**
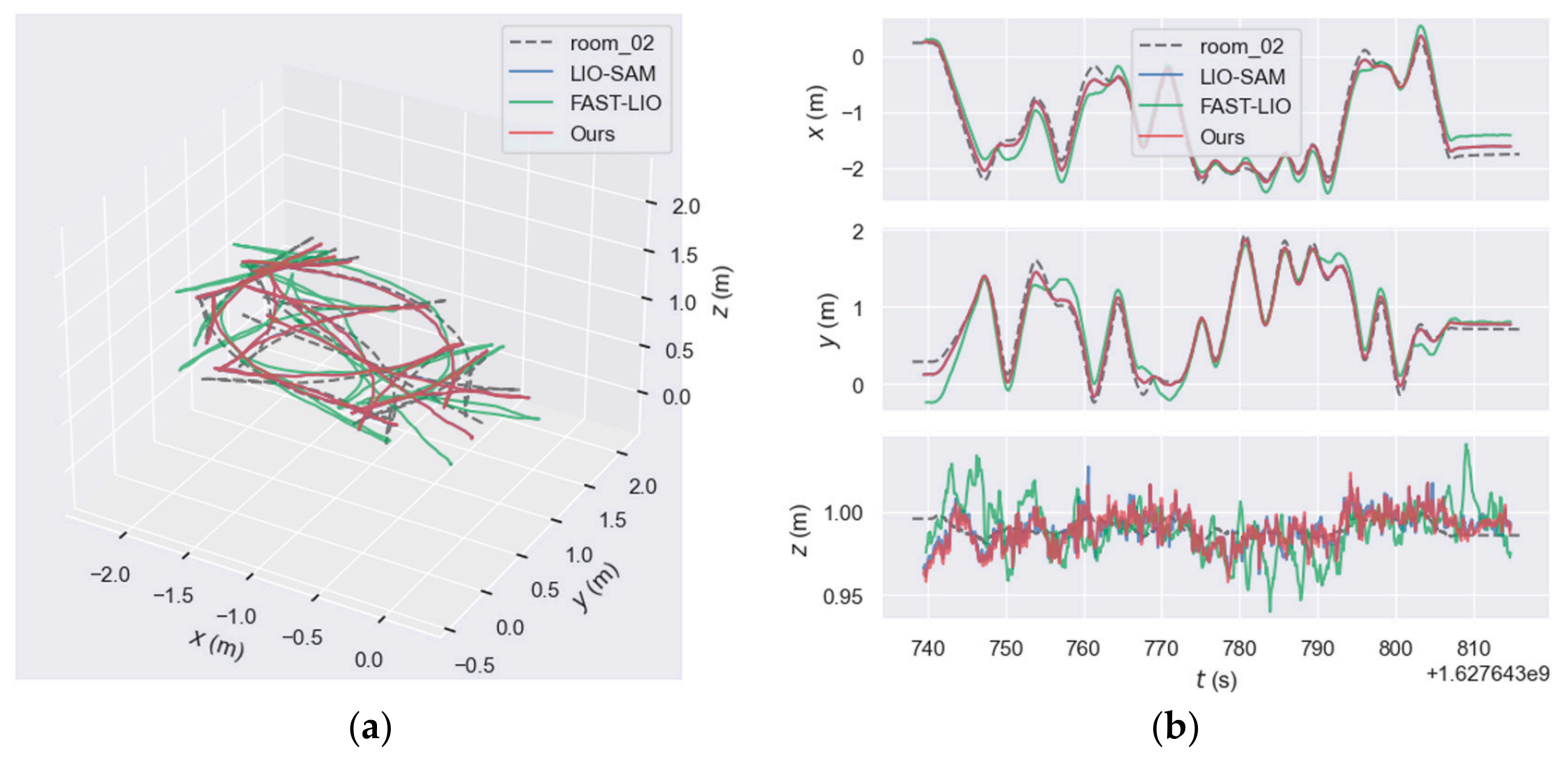
Trajectory comparison results on the M2DGR dataset. (**a**) Trajectory comparison of the selected sequence. (**b**) Coordinate-wise trajectory comparison.

The trajectory comparison on the M2DGR dataset is shown in Figure 8. This experiment evaluates whether the front-end and back-end SLAM backbone can provide usable trajectory estimation under general ground-robot motion conditions.

The quantitative odometry reference results on M2DGR are listed in Table 2, where FAST-LIO and LIO-SAM are used as representative LiDAR-inertial reference methods [7,21].

As listed in Table 2, the M2DGR results are used as reference evidence for the trajectory estimation capability of the SLAM backbone. Since the proposed contributions focus on indoor map refinement and height-aware map encoding, these results are not interpreted as a direct state-of-the-art odometry benchmark.

**Figure 9:**
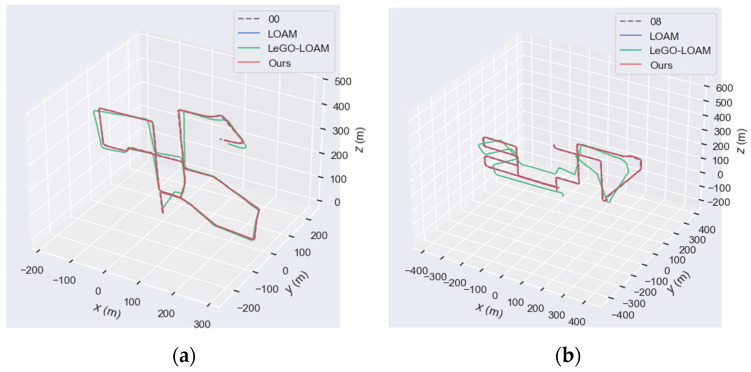
Trajectory comparison results on the KITTI dataset. (**a**) Trajectory comparison of sequence 00. (**b**) Trajectory comparison of sequence 08.

The trajectory comparison on the KITTI dataset is shown in Figure 9.

The quantitative odometry reference results on KITTI are listed in Table 3, where LOAM and LeGO-LOAM are used as representative LiDAR SLAM reference methods [5,6].

As listed in Table 3, the KITTI results provide additional reference evidence for the trajectory estimation capability of the SLAM backbone. The asterisked LeGO-LOAM result on KITTI sequence 00 denotes a divergence case observed under the same initialization, coordinate transformation, timestamp association, and ATE evaluation script used for the other KITTI runs; therefore, this value is retained to explicitly report the failure case rather than being treated as a normal successful run. Because the KITTI sensor suite and motion conditions differ from the proposed indoor robot platform, these results are not used to support claims about dynamic artifact removal or 24-bit height-aware map encoding.

Overall, the public dataset experiments show that the SLAM backbone can provide trajectory estimates suitable for subsequent map construction. The main contributions of this work are evaluated in the following self-collected indoor experiments, where the proposed low-cost sensor configuration, dynamic residual artifacts, and height-aware map export process are available.

### 3.2. Embedded Runtime and Resource Evaluation

The embedded platform experiments evaluate whether the SLAM backbone can operate on a resource-constrained indoor robot platform. The system is implemented on the Sunrise X3 embedded processor, and the processing time of the front-end and back-end modules is measured during indoor robot operation.

**Figure 10:**
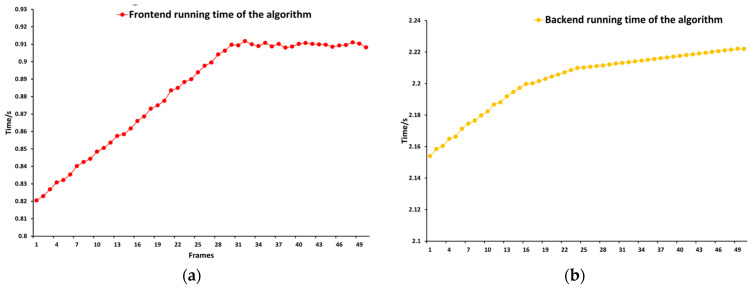
Runtime of the SLAM backbone on the embedded platform. (**a**) Front-end processing time. (**b**) Back-end processing time.

The runtime results in Figure 10 report the module-level processing cost on the embedded platform. According to the implementation, the backend timing is measured only after a keyframe is accepted, and it covers the keyframe-level backend pipeline rather than every incoming sensor frame. Therefore, these results should be interpreted as embedded processing-cost measurements under limited computational resources, rather than as a strict high-frequency real-time guarantee. The front-end and back-end modules require approximately 0.88 s and 2.21 s on average in this test, respectively.

**Figure 11:**
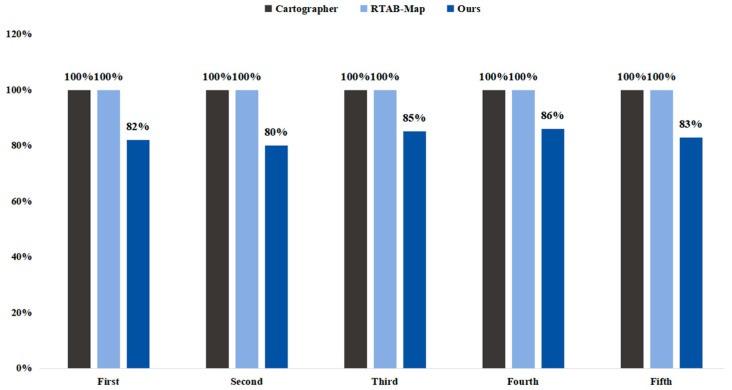
Memory usage comparison on the embedded platform.

The memory usage comparison with representative SLAM systems, including Cartographer and RTAB-Map, is shown in Figure 11 [28,29].

The memory comparison in Figure 11 further indicates that the proposed system maintains relatively low memory usage under the tested embedded configuration. The offline dynamic artifact removal module is executed after global map fusion, and the 24-bit height-aware map export is performed as a map-output stage. Therefore, these two modules are separated from the frame-level odometry update and do not add computation to every incoming sensor frame.

### 3.3. Standard Indoor Mapping Results

Standard indoor mapping experiments are conducted to evaluate the basic map construction capability of the proposed system on the low-cost LDS-ToF-IMU-odometry platform. Following the original experimental setup, two types of indoor scenes are used: an IEC/ASTM 62885-7 coverage test-bed scene and a household environment scene. The household environment contains multiple room-like regions, which are used to evaluate the consistency of the generated occupancy map.

**Figure 12:**
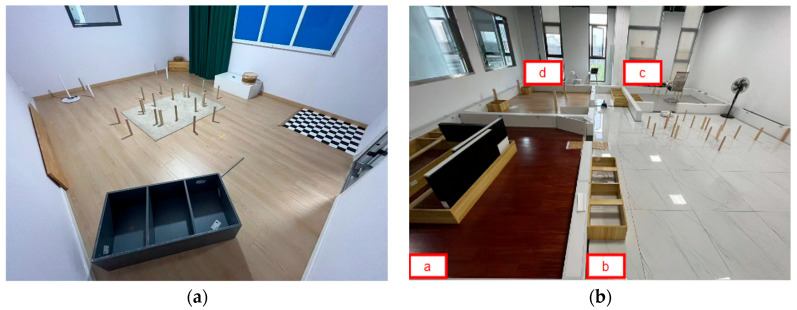
Standard indoor mapping scene. (**a**) IEC/ASTM 62885-7 coverage test-bed scene. (**b**) Household environment scene with multiple room regions.

The tested standard indoor scenes are shown in Figure 12.

**Figure 13:**
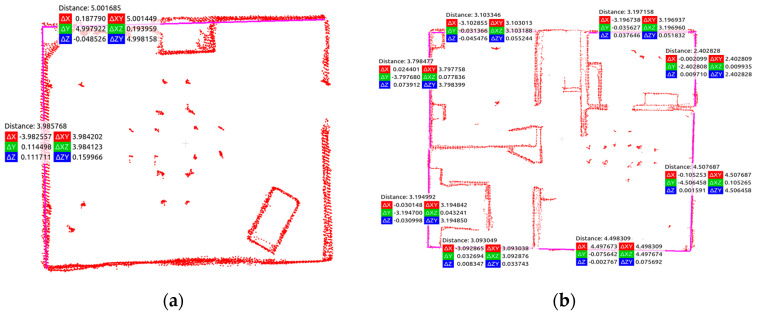
Standard indoor mapping and point cloud measurement results. (**a**) IEC/ASTM 62885-7 coverage test-bed scene; (**b**) household environment scene.

The mapping results of the standard indoor scenes are shown in Figure 13.

The reconstructed point cloud maps in Figure 13 preserve the main boundary structures of the standard indoor scenes. The generated map areas are further compared with the measured scene areas in Table 4 to evaluate basic map consistency.

Table 4 reports small area deviations between the generated occupancy maps and the measured indoor scenes. The area errors remain within an acceptable range for indoor mapping, indicating that the SLAM backbone can provide a reliable global map for subsequent dynamic refinement and height-aware occupancy encoding.

As an additional 2D mapping reference, Gmapping was evaluated on the IEC/ASTM 62885-7 coverage test-bed scene using LDS and wheel odometry. After cropping the exported occupancy map to the measured test-bed region, Gmapping produced a mapped area of 19.81 m^2^, corresponding to an area error of 0.95% compared with the measured area of 20.00 m^2^. In the same scene, the proposed method achieved 19.93 m^2^ with an error of 0.4%.

### 3.4. Dynamic Artifact Removal Evaluation

**Figure 14:**
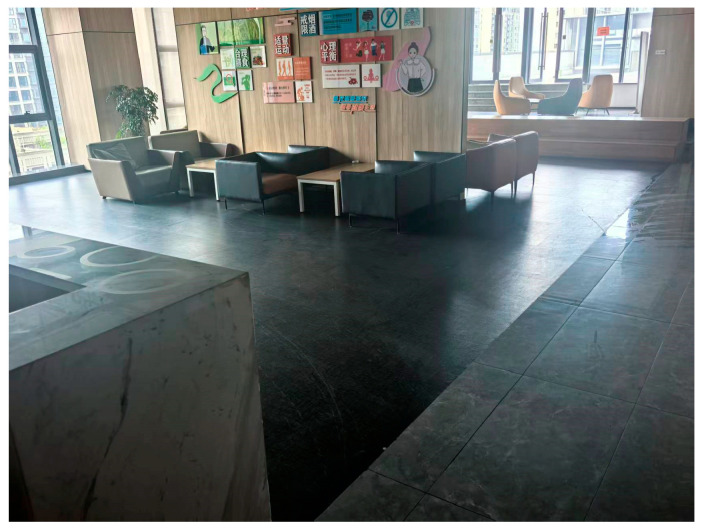
Dynamic loop scene used for artifact removal experiments.

The dynamic artifact removal experiments are conducted in the indoor loop scene shown in Figure 14. Both the long-loop and short-loop sequences are collected in this scene, but with different robot traversal lengths and loop structures. The long-loop sequence contains more repeated observations and visible footprint-like residual artifacts, while the short-loop sequence is used to evaluate the generality of the proposed refinement module under a shorter traversal.

These experiments evaluate whether the proposed offline map refinement module can remove footprint-like residuals, transient human-related artifacts, and sparse ToF artifacts while preserving stable static structures.

#### 3.4.1. Experimental Configuration and Ablation Modes

The dynamic artifact removal experiments are conducted using fixed threshold parameters defined in a configuration file. The same parameter configuration is used for all self-collected indoor sequences unless otherwise stated. The complete parameter list is provided in Appendix A for reproducibility.

The four ablation modes defined in Section 2.7 are used in the following experiments. Mode 0 directly uses the fused global map without cleanup. Mode 1 removes candidate clusters using only geometric constraints. Mode 2 combines temporal voxel support with geometric cluster filtering. Mode 3 further removes sparse isolated low-support points under stricter conditions. This setting allows the contribution of temporal voxel support, geometric constraints, and isolated-point refinement to be evaluated separately.

The raw sensor acquisition rates are approximately 10 Hz for the LDS, 10 Hz for the ToF module, 300 Hz for the IMU, and 50 Hz for wheel odometry. The “sensor rate” reported in Table 5 denotes the effective input-frame rate during the embedded experiments, while the backend frequency is lower because backend updates are triggered at the keyframe level. The complete Mode 3 configuration was also evaluated in terms of runtime and resource usage, as summarized in Table 5.

The backend runs at the keyframe level, so its processing frequency is lower than the sensor rate. The offline refinement and map export steps are executed after global map fusion and are not part of the frame-level odometry loop. Therefore, the reported timings should be interpreted as embedded processing-cost measurements under sparse sensing and keyframe-level processing, rather than as a strict high-frequency real-time SLAM guarantee. CPU usage is computed from process CPU time and may exceed 100% when multiple CPU cores are used.

#### 3.4.2. Long-Loop Scene Ablation

The long-loop scene contains visible footprint-like and transient residual artifacts caused by repeated indoor robot motion. It is used to evaluate the proposed refinement module and the contribution of each cleanup component.

**Figure 15:**
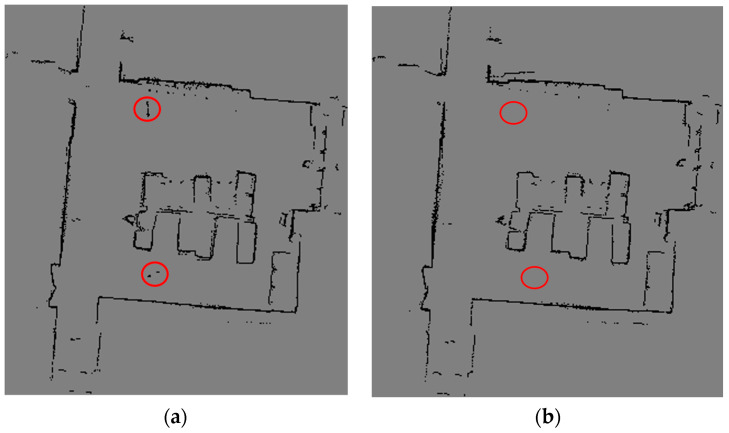
Local 2D occupancy grid comparison before and after dynamic artifact removal in the long-loop scene. (**a**) Occupancy grid generated from the original fused global map; (**b**) occupancy grid generated after dynamic artifact removal. Red circles indicate residual artifacts suppressed after refinement.

The effect of dynamic artifact removal on the final 2D occupancy grid is shown in Figure 15.

After refinement, the isolated residual artifacts marked in Figure 15 are suppressed, while the main wall boundaries and room structures remain preserved.

**Figure 16:**
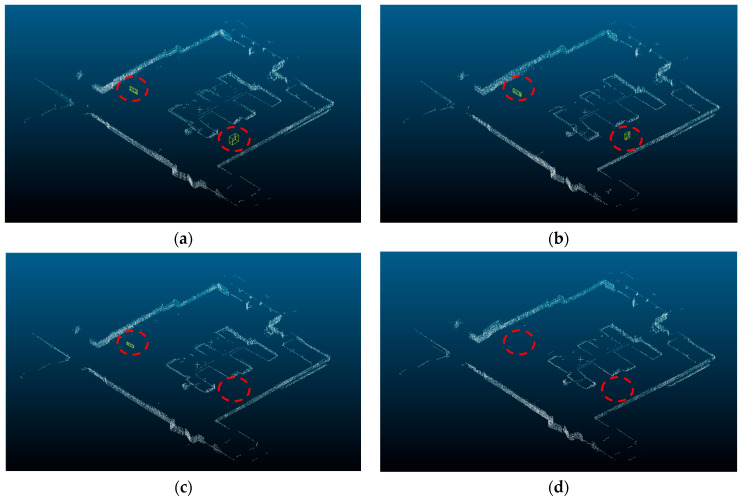
Local comparison of dynamic artifact removal in the long-loop scene. (**a**) Baseline; (**b**) geometry-only filtering; (**c**) temporal support + geometry; (**d**) full method. Red circles indicate footprint-like residual artifacts.

The local ablation comparison is shown in Figure 16.

The quantitative ablation results are listed in Table 6.

To provide a local quantitative reference for Figure 16, residual points were counted in the two marked artifact regions of interest (ROIs). The remaining visible residual points were manually segmented from the corresponding point-cloud results, and the counts are listed in Table 7.

The ROI results provide a local quantitative supplement to the visual comparison. In the two marked ROIs, the number of visible residual artifact points decreases from 83 in the baseline map to 0 after the full refinement, corresponding to an ROI-scoped residual reduction rate of 100%. Geometry-only filtering produces only a small reduction in residual points, whereas temporal voxel support substantially reduces the marked residual regions. With isolated-point refinement enabled, the full method removes the remaining visible residuals in the selected ROIs. Since dense full-scene point-level ground truth is not available for the self-collected dynamic sequence, this ROI-based count is not treated as a full-scene precision, recall, or F1 evaluation.

#### 3.4.3. Cross-Scene Validation

To further evaluate the generality and conservativeness of the proposed refinement module, the full method is tested on three sequences: the long-loop sequence, the short-loop sequence, and the IEC/ASTM 62885-7 coverage test-bed scene. The long-loop and short-loop sequences are collected in the same dynamic loop scene shown in Figure 14, but with different robot traversal lengths and loop structures. The IEC/ASTM test-bed scene is nearly static and is used to evaluate whether the refinement module preserves stable structures when dynamic residual artifacts are weak.

The quantitative cross-scene validation results are listed in Table 8.

Table 8 summarizes the behavior of the full method across different scenes. In the long-loop and short-loop sequences, the method removes residual clusters and isolated points. In the nearly static IEC/ASTM test-bed scene, 323 candidate points are detected, but no clusters or isolated points are removed. To further quantify static-structure preservation in this scene, a preservation rate was computed as the ratio between the output and input point counts after refinement. Under the full method, 10,028 out of 10,028 points were retained, corresponding to a static-structure preservation rate of 100%. Equivalently, no stable point was incorrectly removed in this nearly static scene, corresponding to a static-structure false-removal rate of 0% under this validation setting. This result suggests that the refinement strategy remains conservative when dynamic residual artifacts are weak. The scene contains stable small structures such as table legs, door frames, and low obstacles, providing an additional check that the refinement does not aggressively remove persistent static structures. This result does not imply zero false removal in all environments, but it supports the conservativeness of the selected thresholds in the tested static scene.

### 3.5. 24-Bit Height-Aware Occupancy Encoding Results

The IEC/ASTM 62885-7 coverage test-bed scene is used as the representative case for evaluating the proposed 24-bit height-aware occupancy encoding. This scene contains clear indoor boundaries and local vertical structures, making it suitable for comparing standard 2D occupancy representation, 2.5D height-aware mapping, 24-bit image encoding, and local decoding results.

**Figure 17:**
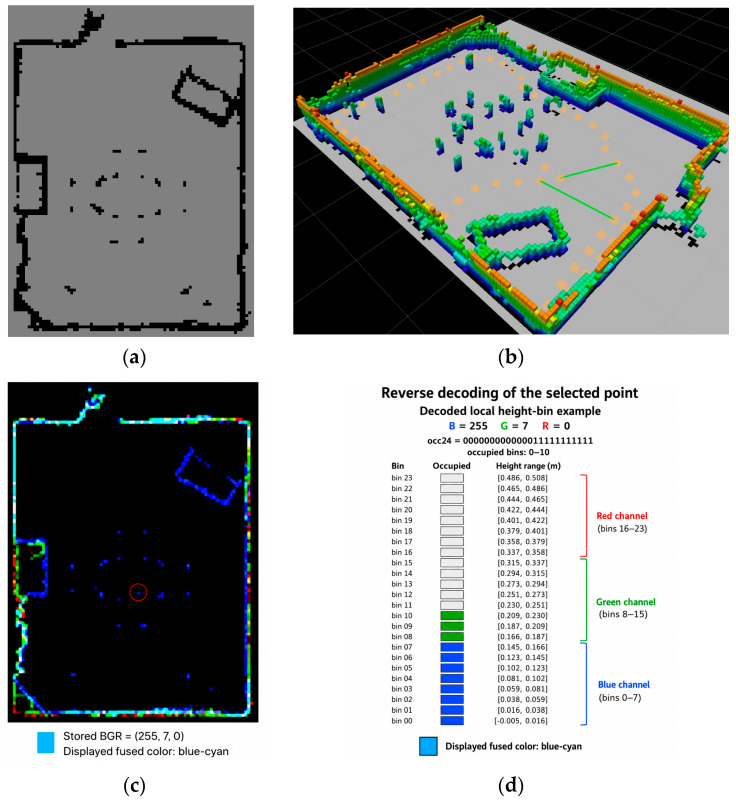
The 24-bit height-aware occupancy encoding result in the IEC/ASTM test-bed scene. (**a**) Standard 2D occupancy grid; (**b**) 2.5D height-aware map; (**c**) encoded 24-bit height-aware map with a selected middle-pillar grid cell marked; (**d**) reverse decoding of the selected grid cell. The selected cell has stored BGR values of (255,7,0), corresponding to occupied height bins from bin 0 to bin 10.

The 24-bit height-aware occupancy encoding result is shown in Figure 17.

Figure 17 compares the standard 2D occupancy grid, the 2.5D height-aware map, the encoded 24-bit map, and the reverse decoding result. The 2D occupancy grid only provides planar occupancy, whereas the 2.5D height-aware map retains additional vertical structure information. The same height-aware occupancy information is compactly packed into the three-channel 24-bit image shown in Figure 17c.

The selected middle-pillar grid cell is decoded in Figure 17d. Its stored BGR values are (255,7,0), corresponding to the 24-bit occupancy value 000000000000011111111111. This value represents occupied height bins from bin 0 to bin 10, while bins 11 to 23 remain unoccupied. The encoded map is saved through a lossless 8-bit three-channel PNG path, so the stored bit pattern of each pixel is preserved. The reverse-decoding example confirms that the saved values can be decoded back to the occupied height bins. This encoding is lossless for the discretized 24-bin occupancy state, but it does not preserve the original continuous 3D point coordinates.

The storage cost of the map formats directly exported by the proposed system was further compared, including the raw PCD map, the conventional 2D occupancy PNG, and the proposed 24-bit height-aware PNG. The results are listed in Table 9. The 24-bit PNG requires more storage than a planar 2D occupancy PNG because it encodes vertical occupancy bins, but it remains much smaller than the raw PCD map. This result shows that the saved PNG is not simply a pseudo-color visualization image, but a decodable data image in which vertical occupancy information can be recovered through bitwise decoding. Unlike downward-filled elevation maps, the proposed representation only encodes directly observed occupied height bins, allowing local vertical structures to be preserved in a compact image-compatible format. After decoding, the height-bin occupancy can be used as an auxiliary height-aware layer for obstacle checking or downstream navigation modules. In this work, the representation is mainly evaluated as a compact map export format, while tighter integration with planning layers is left for future work.

The 24-bit PNG requires more storage than a planar 2D occupancy PNG because it encodes vertical occupancy bins, but it remains much smaller than the raw PCD map.

The proposed 24-bit PNG is not theoretically more compact than a raw 24-bit bitmask with the same grid size. Its advantage lies in standard image-format compatibility, direct inspection, OpenCV-based loading and decoding, and much smaller storage than raw point-cloud export. Therefore, the 24-bit PNG should be interpreted as a compact and deployable height-aware export format rather than as a theoretically minimal map representation.

This result shows that the saved PNG is not simply a pseudo-color visualization image, but a decodable data image in which vertical occupancy information can be recovered through bitwise decoding. Unlike downward-filled elevation maps, the proposed representation only encodes directly observed occupied height bins, allowing local vertical structures to be preserved in a compact image-compatible format.

## 4. Discussion

The experiments indicate that the proposed system can generate usable indoor maps using a low-cost LDS-ToF-IMU-odometry platform. The public datasets are retained only as odometry reference experiments, while the main evaluation is conducted on self-collected indoor scenes that match the proposed sensor configuration. Since the global point cloud is reconstructed from sparse LDS-ToF observations and optimized robot poses, rather than directly acquired from a dense 3D LiDAR, the main contribution of this work lies in compact height-aware map construction and offline map refinement for low-cost indoor robots.

Temporal support plays a central role in the refinement module because transient artifacts and stable structures differ mainly in observation persistence. In the long-loop scene, geometry-only filtering removes only limited artifacts, while temporal voxel support combined with geometric constraints removes more residual clusters. The full method further suppresses sparse isolated low-support points. The cross-scene validation shows that the method remains conservative in the nearly static IEC/ASTM test-bed scene, where stable structures are preserved. Therefore, the refinement result should not be evaluated only by the number of removed points, but by considering both artifact suppression and static-structure preservation.

The 24-bit encoding stores vertical occupancy as height-bin states in a standard three-channel image. Compared with a standard 2D occupancy grid, it preserves occupied height-bin information for each planar grid cell, while remaining more compact than full point cloud storage. The reverse decoding example confirms that the stored BGR values can be converted back into occupied height-bin indices through bitwise operations, showing that the PNG image is a decodable data representation rather than a pseudo-color visualization. What is recovered is the discretized height-bin occupancy, not the original point-level geometry or point density.

This study still has several limitations. The proposed framework is not a new general-purpose SLAM optimizer or dynamic SLAM theory, but a system-level deployment on a low-cost sparse-sensing platform. The public datasets are used as odometry references, while platform-specific mapping and refinement are evaluated on self-collected indoor sequences. The dynamic refinement evaluation relies on ROI-based residual counts and static-scene preservation analysis rather than dense full-scene point-level labels. In addition, the front-end runs online at the keyframe level, whereas refinement and map export are offline post-processing steps; queue latency and power consumption were not separately logged or measured. Finally, the 24-bit encoding preserves discretized height-bin occupancy but not continuous 3D point coordinates. Future work will focus on adaptive height-bin encoding, larger dynamic indoor datasets, parameter adaptation, and tighter integration with downstream robot planning modules.

## 5. Conclusions

This paper presents a lightweight 2.5D SLAM framework for resource-constrained indoor robots using a low-cost LDS-ToF-IMU-odometry sensor configuration. The system reconstructs a global point cloud from sparse multi-sensor observations and optimized robot poses and generates compact height-aware indoor maps.

An offline dynamic artifact removal module is introduced to improve the final map quality. By combining temporal voxel support with geometric constraints, the proposed method suppresses footprint-like residual artifacts while preserving stable static structures. The ablation and cross-scene results show that temporal support is important for distinguishing transient artifacts from persistent indoor structures.

The paper also proposes a 24-bit height-aware occupancy encoding that stores 24 discretized vertical occupancy bins in a three-channel image. The reverse decoding example verifies that the saved PNG is a decodable data representation rather than a pseudo-color visualization. Compared with a standard 2D occupancy grid, this encoding retains vertical occupancy states while avoiding direct storage of the full point cloud. The recovered information is limited to the discretized height-bin occupancy state. Future work will investigate adaptive height-bin encoding, larger dynamic indoor datasets, and integration with downstream robot planning.

## Figures and Tables

**Table 1:** Dynamic cleanup modes for ablation.

Mode	Description	Purpose
0	No cleanup	Baseline
1	Geometry-only filtering	Evaluate geometric rules
2	Temporal support + geometry	Evaluate temporal support
3	Full method	Evaluate complete refinement

**Table 2:** Reference absolute trajectory error (ATE) results on the M2DGR dataset.

Sequence	Runtime/s	FAST-LIO		LIO-SAM		Ours	
		RMSE	MEAN	RMSE	MEAN	RMSE	MEAN
room_01	72	0.395	0.383	0.179	0.163	**0.148**	**0.144**
room_02	75	**0.295**	**0.281**	1.376	1.364	1.571	1.301
gate_01	172	0.241	0.192	0.229	0.189	**0.109**	**0.095**
circle_01	234	**1.021**	0.980	1.225	1.122	1.061	**0.952**
door_01	461	0.159	0.146	0.263	0.244	**0.133**	**0.122**
door_02	127	**0.301**	**0.264**	0.532	0.449	0.311	0.298

**Table 3:** Reference absolute trajectory error (ATE) results on the KITTI dataset.

Sequence	Runtime/s	LOAM		LeGO-LOAM		Ours	
		RMSE	MEAN	RMSE	MEAN	RMSE	MEAN
00	470	2.049	1.950	* 504.935	* 438.832	**1.742**	**1.665**
06	114	**0.785**	**0.731**	2.315	2.183	1.023	0.991
07	114	0.600	0.541	1.395	1.330	**0.563**	**0.512**
08	422	3.411	3.012	**3.282**	**2.921**	3.325	3.145

**Table 4:** Comparison between generated map area and measured scene area.

Scene	Mapping Area/m^2^	Actual Area/m^2^	Inaccuracy/%
IEC/ASTM 62885-7 coverage test bed configuration scene	19.93	20.00	0.4
Household environment a	9.87	9.92	0.5
Household environment b	20.27	20.70	2.1
Household environment c	7.65	7.68	0.4
Household environment d	11.73	11.78	0.4

**Table 5:** Runtime and resource statistics under the complete Mode 3 configuration.

Scene	Sensor Rate (Hz)	Backend Latency (ms)	Backend Freq. (Hz)	CPU Usage (%)	Peak Memory (MB)	Dynamic Refinement (ms)	PCD Export (ms)	2D PNG Export (ms)	24-bit PNG Export (ms)
Short loop	9.288	196.746	5.083	145.003	130.0	733.118	149.161	26.885	144.414
Room	9.595	244.167	4.096	173.191	130.0	1213.435	174.867	19.631	143.621
Long loop	9.564	190.743	5.243	155.991	133.3	1118.542	247.216	48.683	235.808

**Table 6:** Ablation results of dynamic artifact removal in the long-loop scene.

Method	Input Points	Output Points	Point Variation	Candidate Points	Candidate Clusters	Removed Clusters	Removed Iso. Points
Baseline	14,948	14,948	0.00%	-	-	-	-
Geometry-only	14,761	14,580	−1.23%	-	48	1	-
Temporal + geometry	14,665	13,992	−4.59%	1780	75	50	0
Full method	14,574	13,834	−5.08%	1860	78	54	13

**Table 7:** ROI-based residual point evaluation in the long-loop scene.

Method	ROI-1 Residual Points	ROI-2 Residual Points	Total Residual Points	Reduction
Baseline	50	33	83	0.0%
Geometry-only	48	30	78	6.0%
Temporal + geometry	32	0	32	61.4%
Full method	0	0	0	100.0%

**Table 8:** Cross-scene validation of the full dynamic artifact removal method.

Scene	Method	Input	Output	Variation	Candidate	Removed Clust.	Removed Iso.
Long-loop	Base	14,948	14,948	0.00%	-	-	-
Long-loop	Full	14,574	13,834	−5.08%	1860	54	13
Short-loop	Base	9222	9222	0.00%	-	-	-
Short-loop	Full	9115	8690	−4.66%	1899	25	8
IEC/ASTM test-bed	Base	10,643	10,643	0.00%	-	-	-
IEC/ASTM test-bed	Full	10,028	10,028	0.00%	323	0	0

**Table 9:** Storage comparison of exported map formats.

Scene	PCD Size (KB)	2D PNG Size (KB)	24-bit PNG Size (KB)	2D Map Size	Height Bins
Short loop	298.1	2.18	5.19	293 × 205	24
Room	353.7	0.98	3.46	87 × 153	24
Long loop	494.8	3.84	8.66	312 × 356	24

## Data Availability

Data is contained within the article.

## Appendix A

The offline dynamic artifact removal module uses fixed threshold parameters defined in the configuration file. These parameters are used to control temporal voxel support estimation, low-support candidate extraction, geometric artifact filtering, and trajectory-near isolated point removal. The same configuration is used for all self-collected indoor sequences unless otherwise stated.

**Table A1:** Configuration parameters for offline dynamic artifact removal.

Parameter	Value	Description
flag_clean_dynamic_artifacts	true	Enable offline dynamic artifact removal
dynamic_cleanup_mode	3	Full cleanup mode, including temporal support, geometric filtering, and isolated-point removal
temporal_support_voxel_size	0.10 m	Voxel size for temporal support statistics
temporal_support_min_frames	2	Minimum number of supporting keyframes for a voxel to be regarded as stable
temporal_support_low_ratio_th	0.70	Low-support ratio threshold for candidate artifact removal
temporal_support_tof_only_ratio_th	0.70	ToF-only ratio threshold for removing ToF-dominated residual artifacts
temporal_support_max_lds_ratio_th	0.15	Maximum stable LDS-support ratio allowed for removable candidates
temporal_support_max_time_span_s	1.0 s	Maximum temporal span threshold for short-term observations
temporal_support_short_time_ratio_th	0.85	Short-time observation ratio threshold
trajectory_cleanup_radius	0.20 m	Radius around the robot trajectory for isolated low-support point cleanup
trajectory_strict_low_ratio_th	0.80	Strict low-support ratio threshold for trajectory-near isolated points
trajectory_isolated_remove_radius	0.10 m	Radius used for isolated point removal near the robot trajectory
artifact_cluster_tolerance	0.10 m	Euclidean clustering distance threshold for artifact candidates
artifact_cluster_min_size	8	Minimum size of candidate artifact clusters
artifact_cluster_max_size	60	Maximum size of removable artifact clusters
artifact_max_xy_span	0.22 m	Maximum horizontal span of removable artifact clusters
artifact_min_z_span	0.25 m	Minimum vertical span of candidate artifact clusters
artifact_max_z_span	1.20 m	Maximum vertical span of removable artifact clusters

The cleanup mode is set to 3 in the experiments. In this mode, the module first estimates temporal support for map voxels, then removes low-support candidate clusters that satisfy the geometric artifact constraints, and finally removes sparse isolated low-support points near the robot trajectory under stricter thresholds. This conservative configuration is designed to suppress transient residual artifacts while preserving stable structures observed by LDS measurements.

## Appendix B

To check whether the refinement result is strongly affected by the selected thresholds, two key parameters were varied around the current configuration on the long-loop sequence. Only one parameter was changed at a time, while all other settings were kept fixed.

**Table A2:** One-at-a-time parameter perturbation results.

Parameter	Value	Removed Points	Removed Ratio (%)	Removed Clusters	Runtime (ms)
temporal_support_min_frames	1	15	0.104	1	1100.190
temporal_support_min_frames	2	32	0.221	2	1136.894
temporal_support_min_frames	3	95	0.654	5	1135.673
artifact_cluster_tolerance	0.08	42	0.292	3	1012.473
artifact_cluster_tolerance	0.10	32	0.221	2	1136.894
artifact_cluster_tolerance	0.12	36	0.240	2	1128.335

The removed-point ratio remained below 1% in all tested settings. For temporal_support_min_frames, increasing the value from 1 to 3 led to a higher removal ratio, because stricter temporal support rejects more short-lived observations. For artifact_cluster_tolerance, the removal ratio changed only slightly within the tested range from 0.08 m to 0.12 m. Overall, the refinement behavior remains conservative around the tested configuration. This analysis is intended to examine local parameter robustness around the selected configuration rather than to provide a complete generalization study across all possible scenes, robot speeds, keyframe densities, and ToF fields of view.
